# Supplementary material for: Genome-wide meta-analyses of non-response to antidepressants identify novel loci and potential drugs
Source: Res Sq. 2024 Dec 23:rs.3.rs-5418279. Preprint. [Version 1] doi: 10.21203/rs.3.rs-5418279/v1 (PMC11703334; doi:10.21203/rs.3.rs-5418279/v1)
Supplement: Supplement 1 [file NIHPPRS5418279v1-supplement-1.pdf]

Supplementary Files

This is a list of supplementary files associated with this preprint. Click to download.

- Supplementarymaterial.pdf
- Supplementarytables.xlsx
